# Data on kinetic, energy and emission performance of biodiesel from waste frying oil

**DOI:** 10.1016/j.dib.2018.04.017

**Published:** 2018-04-10

**Authors:** Silverio Catureba da Silva Filho, Amanda Carvalho Miranda, Thadeu Alfredo Farias Silva, Felipe Araújo Calarge, Roberto Rodrigo de Souza, José Carlos Curvelo Santana, Elias Basile Tambourgi

**Affiliations:** a Industrial Engineering Post-Graduation Program, Nine July University, Sao Paulo, São Paulo, Brazil; b Smart and Sustainable Cities Post-Graduation Program, Nine July University, São Paulo, Brazil; c School of Chemical Engineering, State University of Campinas, Brazil; d Department of Chemical Engineering, Federal University of Sergipe, SE, Brazil

**Keywords:** Biodiesel, Kinetic curves, Greenhouse gas emission, Energy efficiency

## Abstract

The data presented in this article are related to the research article “Environmental and techno-economic considerations on biodiesel production from waste frying oil in São Paulo city” (Silva Filho et al., 2018) [1]. This article presents the variation of the concentration of waste frying oil (WFO) with the reaction time and temperature during the transesterification of WTOs collected in the residences and restaurants of the city of São Paulo. Then, the biodiesel samples were mixed with the S-10 diesel oil in order to obtain the B10, B20, B30, B40, B50, B75 and B100 blends, which were tested in a diesel engine and their power, fuel consumption and gas emissions (CO, CO_2_ and SO_2_) have been measured to verify their greenhouse effect and energy efficiency.

**Specifications Table**

**Table:** 

Subject area	*Environmental Science*
More specific subject area	*Biofuel and energy*
Type of data	*Tables*
How data was acquired	*Gas analyser, energy controller, power generator, liquid-liquid extraction.*
Data format	*Raw and analysed*
Experimental factors	Residence and restaurant waste frying oils *were transesterified with ethyl alcohol under the time and temperature effects and the effects on the energy efficiency and greenhouse gas of seven blends of these biodiesels were verified.*
Experimental features	The relation between the waste frying oil source with the reaction time and temperatures and their biodiesels with the generation of energy and emission of greenhouse gases
Data source location	School of Chemical Engineering of State University of Campinas from Campinas and Industrial Engineering Post Graduation Program of Nine July University *from São Paulo, Brazil*
Data accessibility	The data are available with this article
Related research article	Silva Filho, S. C.; Miranda, A. C.; Silva, T. A.; Calarge, F. A.; Souza, R. R.; Santana, J. C. C.; Tambourgi, E. B. Environmental and techno-economic considerations on biodiesel production from waste frying oil in São Paulo city. J. Clean. Prod., 183, 1034 – 1042, 2018.

**Value of the data**

• Data on transesterification kinetic also is useful to determine time temperature and composition to obtain high-quality biodiesel from waste frying oil;
• Data on Biodiesel greenhouse gases emission is useful to compare to emission of other fuels;
• Data on biodiesel energy efficiency is a good indicator to choose the best biodiesel blends to use in power generation.

## 1 Data

The data showed in this article is related to the research article by Silva Filho et al. [1], which made comparison energy and gas emission efficiencies between biodiesel blend and diesel oil. Biodiesel was obtained from waste frying oil from restaurant and residences of city of São Paulo, Brazil.

## 2 Experimental design, materials, and methods

This analysis was performed in a jacketed reactor and 100 mL total volume, into which we added 50 mL of oil and 8.33 mL of alcohol and mixed them in the reactor (6:1 volumetric ratio). The reactions proceeded at temperatures of 40 °C, 50 °C, 60 °C, 70 °C, and 80 °C, with 0.1% NaOH (m/v) as a catalyst during constant stirring for up to 120 min. Measurement of the biodiesel amount formed was carried out every 10 min by decanting into a separating funnel; the light phase was washed with petroleum ether and separated in a Soxhlet extractor [2]. Full data are showed in Table 1, Table 2. After adjusting the kinetic data to a model, the parameters presented in Table 3 were generated and used to calculate the activation energy, assuming a batch stirred tank reactor and zero-order models were tested, by means of the concentration (C_A_) of waste frying oil. This way, adjustments were made to find the kinetic constant (k) and the initial concentration (C_A0_). After determination of the kinetic constants, activation energy (*E*_*a*_) of the transesterification reaction was calculated based on the linearised Arrhenius equation. All fitting model were evaluated of according to [3], [4].

**Table 1:** Data on concentration and yield of the transesterification of the waste frying oil from residences.

Time (min)	40 °C		50 °C		60 °C		70 °C		80 °C	
	C (mol/L)	Yield(%)	C (mol/L)	Yield(%)	C (mol/L)	Yield(%)	C (mol/L)	Yield(%)	C (mol/L)	Yield(%)
0	0.8815	0.000	0.8815	0.000	0.8815	0.000	0.8815	0.000	0.8815	0.000
10	0.7294	17.14	0.8053	8.583	0.8303	5.76	0.6934	21.20	0.7807	11.36
20	0.5456	37.86	0.6579	25.20	0.6395	27.27	0.4703	46.35	0.6377	27.48
30	0.4113	53.00	0.5365	38.89	0.4958	43.48	0.2059	76.15	0.5248	40.21
40	0.2712	68.79	0.4140	52.70	0.3985	54.44	0.1578	81.58	0.0247	96.57
50	0.1548	81.91	0.3048	65.00	0.1777	79.33	0.0247	96.57	0.0262	96.41
70	0.0366	95.23	0.1570	81.67	0.0220	96.88	0.0410	94.73	0.0187	97.26
80	0.0292	96.06	0.0213	96.96	0.0027	99.69	0.0586	92.75	0.0427	94.55
120	0.0001	99.89	0.0396	94.89	0.0058	98.71	0.0476	94.00	0.0108	99.88

**Table 2:** Data on concentration and yield of the transesterification of the waste frying oil from restaurants.

Time (min)	40 °C		50 °C		60 °C		70 °C		80 °C	
	C (mol/L)	Yield(%)	C (mol/L)	Yield(%)	C (mol/L)	Yield(%)	C (mol/L)	Yield(%)	C (mol/L)	Yield(%)
0	0.8815	0.000	0.8815	0.000	0.8815	0.000	0.8815	0.000	0.8815	0.000
10	0.8141	7.600	0.8223	6.072	0.6863	20.04	0.5444	34.61	0.5319	35.89
20	0.7617	13.50	0.7514	13.36	0.6331	25.50	0.4557	43.72	0.3429	55.30
30	0.5089	42.00	0.7218	16.39	0.4645	42.81	0.3048	59.20	0.2664	63.15
40	0.3847	56.00	0.5118	37.95	0.2960	60.12	0.0476	85.61	0.0195	88.50
50	0.2876	66.94	0.1556	74.52	0.0653	83.79	0.0209	88.35	0.0266	87.77
70	0.1777	79.33	0.1174	78.44	0.0209	88.35	0.0209	88.43	0.0105	89.42
80	0.0269	96.33	0.0032	90.51	0.0121	89.26	0.0209	88.50	0.0121	89.26
120	0.0239	96.67	0.0195	88.50	0.0032	90.17	0.0121	89.26	0.0121	89.35

**Table 3:** Data to calculate the activation energy (AE) by Arrhenius method.

T (°C)	Residences frying oil		Restaurants frying oil	
	k (10^−4^ mol/s)	T (K)	k (10^−4^ mol/s)	T (K)
40	1.817	313.15	1.750	313.15
50	2.033	323.15	2.283	323.15
60	2.450	333.15	2.483	333.15
70	2.933	343.15	2.833	343.15
80	3.283	353.15	3.317	353.15

The energy data generated after the combustion of the oil in the generator, as well as fuel consumption and efficiencies are shown in Table 4. The resultant residential and restaurant biodiesels were mixed in these proportions, and their blends in diesel oil ranging from 10% to 100% were tested on a diesel engine. Thus, we conducted a comparison of their energy and greenhouse gas emission efficiency rates in relation to diesel oil marketed in Brazil at the time of the design of this study. These data were used by Silva et al. [1] to calculate the energy generation efficiencies, fuel consumption to determine the best biodiesel blend to be used in electric generators without losing the efficiency of converting the fuel to electric energy.

**Table 4:** Energy efficiency of biodiesel from waste frying oil.

Parameters	Diesel oil	Biodiesel blends (%)						
	Control	10	20	30	40	50	75	100
Power (kW h)	2.95	2.85	2.93	2.95	2.96	2.95	2.98	2.95
Fuel consumption (L/h)	1.33	1.42	1.58	1.52	1.82	2.58	2.71	2.5
Power per Fuel Volume (kW/L)	2.22	2.01	1.85	1.94	1.63	1.14	1.10	1.18

Table 5 shows average values of gases measured in this work. The CO, CO_2_, and SO_2_ amounts were measured every 2 s via insertion of the BEA720 gas analyser sensor (Bosch Ltd®) into the gas outlet duct of the power generator engine. Measurements were taken during power generation using diesel oil and biodiesel blends of 10, 20, 30, 40, 50, 75 and 100% purity. These measurements were used to compare the gaseous emissions of biodiesel samples with the control (diesel oil), as shown in Silva et al. [1].

**Table 5:** Emission efficiency of biodiesel from waste frying oil.

Gas	Diesel oil	Biodiesel blends (%)						
	Control	10	20	30	40	50	75	100
CO (%, v/v)	0.060	0.042	0.044	0.053	0.049	0.031	0.037	0.035
CO (ppm)	0.623	0.438	0.459	0.545	0.512	0.321	0.382	0.539
CO_2_ (%, v/v)	2.707	2.113	2.215	2.434	1.868	1.318	1.328	1.520
CO_2_ (ppm)	44.07	34.40	36.06	39.63	30.41	21.45	21.63	24.75
SO_2_ (%, v/v)	0.127	0.107	0.086	0.078	0.056	0.033	0.016	0.000
SO_2_ (ppm)	3.008	2.535	2.025	1.842	1.319	0.7754	0.369	0.000
