# Supplementary material for: Data on kinetic, energy and emission performance of biodiesel from waste frying oil
Source: Data Brief. 2018 Apr 10;18:1224–8. doi: 10.1016/j.dib.2018.04.017 (PMC5996946; doi:10.1016/j.dib.2018.04.017)
Supplement: Supplementary file 1 — Supplementary material [file mmc1.doc]

Dear Data in Brief Editor,

I, José Carlos Curvelo Santana, in name of all authors (Silvério Catureba da Silva Filho, Amanda Carvalho Miranda, Thadeu Alfredo Farias Silva, Felipe Araújo Calarge e Elias Basile Tambourgi) am re-submitting the manuscript “Data on Kinetic, Energy and Emission Performance of Biodiesel from Waste Frying Oil” to you journal.

We declare that there is no conflict of interest, that the article and the data have never been published in other journals or scientific meetings and that we assign the copyright to the text and data of the aforementioned article to Elsevier.

I’m available to ask your questions.

Thank you.

São Paulo, SP, Brazil, 2018-03-13.

**
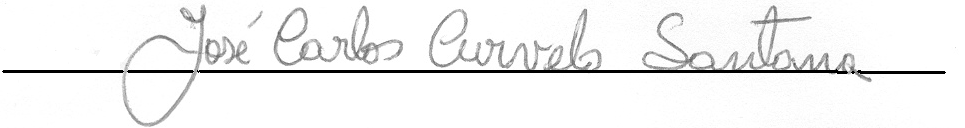
**

Professor DSc, José Carlos Curvelo Santana

Industrial Engineering Postgraduate Programme, UNINOVE, Vergueiro Street, 235/249, Liberdade, Zip Code: 01504-000, São Paulo-SP, Brazil.

E-mail: [jccurvelo@uninove.br](mailto:jccurvelo@uninove.br); [jccurvelo@yahoo.com.br](mailto:jccurvelo@yahoo.com.br)
